# Bone Turnover Markers and Bone Mineral Density to Predict Osteoporotic Fractures in Older Women: A Retrospective Comparative Study

**DOI:** 10.1111/os.12596

**Published:** 2019-12-27

**Authors:** Xiao‐long Qu, Bo Zheng, Tian‐yi Chen, Zong‐rui Cao, Bo Qu, Tao Jiang

**Affiliations:** ^1^ Chengdu Medical College The First Affiliated Hospital of Chengdu Medical College Chengdu China; ^2^ Department of Orthopaedic Surgery The First Affiliated Hospital of Chengdu Medical College Chengdu China

**Keywords:** Bone mineral density, Bone turnover markers, Osteoporotic fractures, Older women

## Abstract

**Objective:**

To investigate the clinical significance of the detection of bone mineral density (BMD) and bone turnover markers (BTM) in older women with osteoporosis, and to compare their predictive power for osteoporotic fractures (OF).

**Methods:**

In this retrospective study, 96 patients with OF and 107 patients with osteoporosis who were hospitalized in the Department of Orthopedics at the First Affiliated Hospital of Chengdu Medical College were examined from October 2017 to February 2019. All selected patients were divided into either the fracture group (96 cases, 47.3%) or the non‐fracture group (107 cases, 52.7%). BMD was measured by dual‐energy X‐ray absorptiometry (DXA). BTM were detected by electrochemical luminescence: aminoterminal propeptide of type I procollagen (PINP), β‐cross‐linked C‐telopeptide of type I collagen (β‐CTX), and molecular fragment of osteocalcin N terminal (N‐MID). Bone metabolism‐related indicators were detected, including alkaline phosphatase (ALP), calcium (Ca), and phosphorus (P). Independent‐samples *t*‐tests were used to compare the measurement data between the two groups, one‐way ANOVA to compare the gaps between groups, and binary logistic regression to analyze the correlation of BMD and BTM with OF.

**Results:**

There were no significant differences in age, weight, height, body mass index, age, and time of menopause between the two groups. There were a total of 71 cases (35.0%) in group A (60–70 years), 80 cases (39.4%) in group B (71–80 years), and 52 cases (25.6%) in group C (81–90 years). The fracture group was compared with the non‐fracture group for BMD in the lumbar (0.75 ± 0.05 *vs* 0.88 ± 0.13, 0.75 ± 0.16 *vs* 0.87 ± 0.09, 0.74 ± 0.21 *vs* 0.87 ± 0.12 g/cm^2^; *P* < 0.05), BMD in the hip (0.62 ± 0.16 *vs* 0.74 ± 0.14, 0.61 ± 0.15 *vs* 0.73 ± 0.0, 0.58 ± 0.13 *vs* 0.73 ± 0.08 g/cm^2^; *P* < 0.05), PINP (83.7 ± 5.7 *vs* 74.8 ± 5.0, 80.7 ± 4.1 *vs* 72.1 ± 5.1, 81.2 ± 7.0 *vs* 68.7 ± 6.3 ng/mL, *P* < 0.05), and β‐CTX (829.7 ± 91.5 *vs* 798.8 ± 52.2, 848.1 ± 71.2 *vs* 812.4 ± 79.0, 867.3 ± 53.1 *vs* 849.1 ± 67.2 pg./mL, *P* < 0.05). N‐MID (19.0 ± 6.7 *vs* 21.3 ± 9.7, 16.2 ± 7.0 *vs* 18.0 ± 5.3 ng/mL, *P* < 0.05) in the fracture cases was lower than in the non‐fracture cases for groups B and C, and there was statistical significance. Among the fracture cases, PINP in group A was higher than in group B and C, and β‐CTX in group C was higher than in group A and B (*P* < 0.05). There was no significant difference in the ALP, P, and Ca between the two groups (*P* > 0.05). Binary logistic regression analysis showed that for BMD in the lumbar and hip, β‐CTX and OF were significantly correlated (respectively, odds ratio [*OR*] = −4.182, 95% confidence interval [*CI*] 1.672–3.448; *OR* = 6.929, 95% *CI* 2.586–12.106; *OR* = 7.572, 95% *CI* 1.441–3.059), and the differences were statistically significant. PINP and N‐MID were correlated with OF (respectively, *OR* = 4.213, 95% *CI* 0.978–1.005; *OR* = 2.510, 95% *CI* 1.070–1.134, *P* > 0.05), the difference was not statistically significant.

**Conclusion:**

Osteoporotic older women, with lower bone density and higher β‐CTX, are more likely to incur OF. β‐CTX is better than BMD at predicting OF and can help in its management and in implementing interventions in high‐risk populations.

## Introduction

Osteoporosis is a bone disease characterized by decreasing bone mass, damage to the bone tissue microstructure, increasing bone brittleness, and an inclination towards fragility fractures. The disease poses a serious threat to older postmenopausal women^1^. Many elderly women are in a high metabolic state as a result of menopause and aging. The decrease of estrogen and the increase of inflammatory mediators during menopause lead to increased osteoclast activity, and osteolysis dominates osteoformation. Older women with osteoporosis may suffer from chronic limb and lumbar back pain, spinal deformity, and fragility fractures caused by low energy trauma, which are common in the spine vertebral body and hip, the proximal humerus, and the distal radius. The number of brittle fractures is increasing year by year, so the tools for predicting osteoporotic fractures (OF) need to be further improved. Currently, OF can be predicted by using bone mineral density (BMD), bone turnover markers (BTM), the WHO Fracture Risk Assessment Tool (FRAX), quantitative CT, and quantitative ultrasound (QUS)^2^, but the accuracy and measurement of various assessment tools are not uniform and there was no study to compare the effectiveness of their assessments. BMD and BTM are not only risk assessment tools but risk factors^3^. However, the usefulness of BTM to predict OF is still in doubt, and the relevant research is not adequate. Social health policy‐makers should implement targeted screening and management programs for osteoporosis as demand for medical care increases worldwide. At the same time, effective intervention measures must be taken to prevent falls, maintain the proportion of targeted medical resources, reduce the number of new patients, and ensure the rationalization of medical resources.

Scholars have suggested that DXA should be used as a tool for the diagnosis of osteoporosis, and the threshold value for the diagnosis of osteoporosis is T value <−2.5 SD^1^. Although race, region, fracture history, hormones, and other factors may affect the occurrence of OF, OF risk assessment based on BMD is also recommended^4^. The bone metabolism biochemical markers include calcium and phosphorus metabolism indicators, bone formation markers, bone resorption markers, hormones, and cytokine. BTM can reflect the state of bone turnover, with high sensitivity and specificity, and has been used to predict fracture risk and for diagnosis of osteoporosis. Bone formation markers include alkaline phosphatase (ALP), bone specific ALP, molecular fragment of osteocalcin N terminal (N‐MID), osteoprotegerin, type I procollagen carboxyl‐terminal peptide (PICP), and aminoterminal propeptide of type I procollagen (PINP). Bone resorption markers include tartrate‐resistant acid phosphatase, N‐telopeptide of type I collagen (NTX), C‐telopeptide of type I collagen (CTX), urinary pyridinoline, and urinary deoxypyridinoline. In addition, PINP and CTX were also recommended as bone formation markers and bone resorption markers, respectively, to predict the osteoporosis progress and the occurrence of OF^5^. N‐MID is an R‐hydroxy glutamic acid protein. An increase of N‐MID concentration indicates that the rate of bone formation is accelerated. The level of N‐MID in serum can directly reflect the osteoblast activity and bone formation in patients with osteoporosis, and it also has some reference value for the dynamic change of blood concentration in patients treated with anti‐osteoporosis drugs. The N‐MID test combined with other bone metabolism indicators has been widely used in the diagnosis of postmenopausal osteoporosis, the monitoring of anti‐bone resorption therapy, and the prediction of fracture risk. Different studies have reported that bone markers are related to the occurrence of hip fractures in the elderly, but many studies are biased, and some studies are not reliable. By comparing and analyzing the relationship between bone density and bone markers, some guidance may be provided for clinical practice.

The latest guide proposed that BTM can be used for OF risk assessment, but the current results of clinical studies are not completely consistent^3^. There is a modest but significant association between BTM and the OF risk. Currently, the International Federation of Clinical Chemistry and Laboratory Medicine (IFCC) and the International Osteoporosis Foundation (IOF) are making efforts to develop the use of BTM for fracture risk prediction. Because BTM are of great significance in the diagnosis, treatment monitoring, and follow‐up observations of osteoporosis, there must be correlation between BTM and OF. At present, there is no large statistical study to clarify the specific relationship between BTM and OF. The problem is being studied worldwide, multi‐dimensional analysis is being conducted through basic research and clinical trials, and reference indicators are expected to be available soon.

In this retrospective study, we tested BMD and BTM in older women with osteoporosis to: (i) explore their ability to predict the OF risk by regression analysis and observe which indicators are more sensitive; (ii) judge the suitability of the two assessment tools and analyze the effects of different markers on OF; (iii) hopefully make to early predict clinical OF and take timely reasonable intervention measures. Finally, we hope that the results of our study can provide a reference for follow‐up studies on osteoporotic fractures as well as guidance for clinical practice.

## Materials and Methods

### *General Data*

From October 2017 to February 2019, 203 patients who were hospitalized in the Department of Orthopedics at the First Affiliated Hospital of Chengdu Medical College were examined. All patients were diagnosed with osteoporosis by using DXA to detect BMD; 96 patients with OF and 107 patients with just osteoporosis were divided into fracture and non‐fracture groups, respectively. Age, menopause age and time, height, weight, and body mass index (BMI) of all patients were collected, and patients were divided into 71 cases in group A (60–70 years old), 80 cases in group B (71–80 years old), and 52 cases in group C (81–90 years old).

### *Inclusion Criteria*

Inclusion criteria: (i) postmenopausal women aged 60–90 years with a diagnosis of osteoporosis; (ii) a portion of patients suffered from fresh fragility fractures including the hip, the vertebra body, the distal radius or the proximal humerus; the fracture group experienced a fall or slipped, while the non‐fracture group also experienced both events; (iii) be able to observe the level of BMD of the lumbar spine and hip, BTM (PINP, β‐CTX, and N‐MID) and bone metabolism‐related indicators (ALP, Ca, and P); and (iv) be able to compare the differences of various indicators between the two groups using statistical methods and to analyze the significance of their representatives.

### *Exclusion Criteria*

Exclusion criteria: (i) patients with severe heart, lung, and kidney diseases who cannot tolerate relevant examinations; (ii) those with hyperthyroidism, Cushing's syndrome, multiple myeloma, or Paget's bone disease; (iii) long‐term use of hormones, chemotherapy drugs, or aromatase inhibitors; (iv) fractures caused by high trauma such as traffic accidents and high fall injuries and patients who have undergone previous anti‐osteoporosis treatment; (v) previous history of fragility fractures; (vi) and pathological fractures caused by metastatic or primary bone tumors.

### *Study Methods*

General information collected for all subjects included: (i) BMD of the lumbar spine and hip; (ii) BTM: PINP, β‐CTX, and N‐MID; (iii) bone metabolism‐related indicators, ALP, Ca, and P. BMD was were measured using DXA (enCORE, General Electric, version 15), instrument quality control was carried out before measurement, and those subjects with T < −2.5 SD were diagnosed as having osteoporosis according to diagnosis criteria^1^. BMDL and BMDH represent BMD of the lumbar and the total hip, respectively. All subjects fasted (water and food) from 22:00 hours on the first inpatient day, and 5 mL of fasting blood was extracted at 06:00 hours the next morning. All the samples were sent to the medical laboratory of the hospital for determination of bone metabolism‐related indicators and BTM. ALP, P, and Ca were determined using common biochemical methods; PINP, β‐CTX, and N‐MID were measured by electrochemical luminescence. The kit was provided by Roche, the samples were centrifuged at 1006.2 g for 5 min, the upper serum was taken and next measured using Cobas e411 automatic analyzer produced by Roche Company, and the data were read after quality control.

### *Statistical Analysis*

All data were analyzed using IBM SPSS statistics 21.0. The measurement data between the two groups were tested by independent samples *t*‐test and expressed as mean plus or minus standard deviation (mean ± SD). One‐way ANOVA was conducted to compare the gap between groups. Binary logistic regression was used for the correlation analysis and the *OR* indicated the correlation degree. *P* < 0.05 was considered a statistically significant difference.

## Results

### *Comparison of General Data*

The differences were not statistically significant for age (74.8 ± 10.0 *vs* 71.5 ± 6.1), weight (157 ± 5.1 *vs* 155 ± 4.8), height (55.8 ± 6.6 *vs* 55.1 ± 5.3), BMI (22.4 ± 3.5 *vs* 24.0 ± 4.7), age, and time of menopause (51.4 ± 4.6 *vs* 50.2 ± 4.1 and 23.4 ± 5.9 *vs* 21.3 ± 4.2) when comparing the fracture group with the non‐fracture group (*P* > 0.05). Demographic information was comparable, as shown in Table 1.

**Table 1:** Comparison of general data between two groups (mean ± SD)

Indexes	Fracture group (96 case)	Non‐fracture group (107 cases)	*P*‐value
Age (years)	74.8 ± 10.0	71.5 ± 6.1	0.11
Height (cm)	157.0 ± 5.1	155.0 ± 4.8	0.172
Weight (kg)	55.8 ± 6.6	55.1 ± 5.3	0.148
BMI (kg/m^2^)	22.4 ± 3.5	24.0 ± 4.7	0.135
Age of menopause (years)	51.4 ± 4.6	50.2 ± 4.1	0.158
Time of menopause (years)	23.4 ± 5.9	21.3 ± 4.2	0.141

After the *t*‐test analysis, there was no significant difference between the fracture group and the non‐fracture group (*P* > 0.05).

#### *Comparison of bone mineral density and bone turnover markers*

BMD, PINP, β‐CTX, N‐MID, ALP, Ca, and P were compared for the fracture group and the non‐fracture group. BMDL and BMDH in the fracture group were lower than in the non‐fracture group for all age groups; the differences were statistically significant (*P* < 0.05). Comparing BMD in all age groups within the fracture group and the non‐fracture group, there was no statistical significance (*P* > 0.05). PINP and β‐CTX were higher in the fracture group for all age groups. N‐MID was lower in the fracture group for those aged 70–90 years; the differences were statistically significant (*P* < 0.05). In group A, PINP within the fracture group was higher than in groups B and C (*P* < 0.05). In group C, β‐CTX between fracture and non‐fracture groups was higher than for groups A and B (*P* < 0.05), and there was statistical significance, as shown in Tables 2 and 3. ALP, P, and Ca show no significant difference between the fracture group and the non‐fracture group, but there was significance difference within groups (*P* > 0.05), as shown in Table 4.

**Table 2:** Comparison of BMDL and BMDH between two groups (mean ± SD)

Groups	BMDL (g/cm^2^)			One‐way ANOVA	BMDH (g/cm^2^)			One‐way ANOVA
	A	B	C	*P*‐value	A	B	C	*P*‐value
Fracture group	0.75 ± 0.05	0.75 ± 0.16	0.74 ± 0.21	>0.05	0.62 ± 0.16	0.61 ± 0.15	0.58 ± 0.13	>0.05
Non‐fracture group	0.88 ± 0.13	0.87 ± 0.09	0.87 ± 0.12	>0.05	0.74 ± 0.14	0.73 ± 0.06	0.73 ± 0.08	>0.05
*P‐*value	0.001	0.018	0.002		0.002	0.01	0.006	

Comparing all age groups by one‐way ANOVA within the fracture group and the non‐fracture group (*P* > 0.05), there was no statistical significance. Bone mineral density of the lumbar (BMDL) and bone mineral density of the total hip (BMDH) in the fracture group were lower than in the non‐fracture group for all age groups; the differences were statistically significant based on a *t*‐test (*P* < 0.05).

**Table 3:** Comparison of PINP, β‐CTX, and N‐MID between two groups (mean ± SD)

	PINP (ng/mL)				β‐CTX (pg/mL)				N‐MID (ng/mL)			
Groups	A	B	C	*P*‐value	A	B	C	*P*‐value	A	B	C	*P*‐value
Fracture group	83.7 ± 5.7	80.7 ± 4.1	81.2 ± 7.0	<0.05	829.7 ± 91.5	848.1 ± 71.2	867.3 ± 53.1	<0.05	21.6 ± 5.2	19.0 ± 6.7	16.2 ± 7.0	>0.05
Non‐fracture group	74.8 ± 5.0	72.1 ± 5.1	68.7 ± 6.3	>0.05	798.8 ± 52.2	812.4 ± 79.0	849.1 ± 67.2	<0.05	24.3 ± 10.1	21.3 ± 9.7	18.0 ± 5.3	>0.05
*P*‐value	0.01	0.014	0.019		0.02	0.028	0.017		0.058	0.008	0.016	

Based on the *t*‐test and one‐way ANOVA, aminoterminal propeptide of type I procollagen (PINP) and β‐cross‐linked C‐telopeptide of type I collagen (β‐CTX) were higher in the fracture group for all age groups and molecular fragment of osteocalcin N terminal (N‐MID) was lower in the fracture group for groups B and C; the differences were both statistically significant (*P* < 0.05); PINP in A within the fracture group was higher than for B and C (*P* < 0.05); β‐CTX in C between the fracture group and the non‐fracture group was higher than A and B (*P* < 0.05), and there was statistical significance.

**Table 4:** Comparison of ALP, Ca, and P between two groups (mean ± SD)

	ALP (mmol/L)				P (mmol/L)				Ca (mmol/L)			
Groups	A	B	C	*P*‐value	A	B	C	*P*‐value	A	B	C	*P*‐value
Fracture group	98.2 ± 5.0	94.2 ± 5.1	90.9 ± 6.0	>0.05	1.2 ± 0.7	1.2 ± 0.4	1.0 ± 0.1	>0.05	2.1 ± 0.3	2.0 ± 0.7	2.2 ± 0.3	>0.05
Non‐Fracture group	97.6 ± 6.7	99.4 ± 8.7	99.6 ± 9.6	>0.05	1.1 ± 0.3	1.3 ± 0.4	2.0 ± 0.1	>0.05	2.2 ± 0.2	2.1 ± 0.1	2.2 ± 0.5	>0.05
*P*‐value	0.428	0.559	0.416		0.241	0.2	0.307		0.427	0.433	0.27	

Alkaline phosphatase (ALP), calcium (Ca), and phosphorus (P) show no significant difference between the fracture group and the non‐fracture group, also within groups (*P* > 0.05).

### *Correlation of Bone Mineral Density and Bone Turnover Markers and Osteoporotic Fractures*

Binary logistic regression was used to analyze BMDL and BMDH and BTM were used to predict OF risk. BMDL, BMDH, and β‐CTX were significantly correlated with the occurrence of OF (respectively, odds ratio [*OR*] = −4.182, 95% *CI* 1.672–3.448; *OR* = 6.929, 95% *CI* 2.586–12.106; *OR* = 7.572, 95% *CI* 1.441–3.059); β‐CTX had the highest correlation with the risk of OF, and the differences were statistically significant (*P* < 0.05). This revealed that patients with higher β‐CTX and lower BMD were prone to incurring OF. PINP and N‐MID were positively correlated to OF risk (respectively, *OR* = 4.213, 95% *CI* 0.978–1.005; *OR* = 2.510, 95% *CI* 1.070–1.134, *P* > 0.05), but the difference was not statistically significant (*P* > 0.05), as shown in Table 5.

**Table 5:** Binary logistic regression analysis of risk factors for OF

Variables	*β*	SE	Wald	*P* value	*OR*	95% *CI*
BMDL	−3.874	−2.18	−3.159	0.01	−4.182	1.672–3.448
BMDH	−5.176	−2.156	−5.763	0.016	−6.292	2.586–12.106
PINP	2.17	3.182	2.85	0.061	4.213	0.978–1.005
N‐MID	3.101	2.561	3.047	0.08	2.51	1.070–1.134
β‐CTX	3.295	2.703	4.002	0.001	7.572	1.441–3.059

BMDL, BMDH, and β‐CTX were the risk factors of osteoporotic fractures (OF) (*P* < 0.05); the correlation of the occurrence of OF was statistically significant. β‐CTX was most correlated to the risk of osteoporotic fractures (*OR* = 7.572, 95% *CI* 1.441–3.059).

## Discussion

The world population has gradually increased with the reduction of the mortality rate of newborn babies and the lengthening of lifespans. Most countries are facing an aging population. Osteoporosis is among the most common systemic chronic diseases, and poses a serious health threat to older women. A meta‐analysis showed that the prevalence of osteoporosis in middle‐aged and elderly people in China was 23% on average, with 27% of women and 16% of men severally affected; osteoporosis is found to be positively correlated with age^6^. Another study indicated that the prevalence of osteoporosis in older people in China from 2010 to 2016 was 36%: 23% were men and 49% were women^7^. Unfortunately, the high prevalence of osteoporosis is accompanied by a low awareness rate. In general, in most patients, osteoporosis is not diagnosed until severe complications are encountered, such as OF. OF most commonly occur in the hip, distal radius, proximal humerus and vertebral body, related medical expenses due to fracture were in an exponential increase^8^, ^9^, ^10^. It is estimated that in China there will be 4.83 million cases of OF patients by 2035 and 5.99 million cases by 2050^11^. Therefore, understanding the risks and means of preventing OF has become an important objective of public health research.

Currently, BMD, BTM, FRAX tools, the Garvan nomogram evaluation method, and quantitative bone ultrasound are used to predict OF^12^. Although use of BMD to predict OF is the current consensus in academia, there are still some limitations and uncertainties. Patients with low BMD based on clinical observation do not necessarily incur fragility fractures; however, some patients with relatively high BMD incur fragility fractures. With aging and menopause, BTM has shown slight changes before BMD changes, and it can even reflect the changing trend of BMD. Therefore, it may be more appropriate and sensitive for use in predicting OF.

Gossiel *et al*. found that the CTX and PINP levels in postmenopausal women were higher than in premenopausal women (*P* < 0.001), and increased, respectively, 80% in CTX and 33% in PINP within 10 years after menopause; this reinforced that bone loss was significantly correlated with overall bone conversion^13^. A TRIO study by Diez‐Perez *et al*. showed that PINP and CTX were measured at baseline and at 3 months after oral bisphosphonate therapy, and follow‐up treatment management was determined by examining the least significant change (reduction of PINP and CTX by more than 38% and 56%, respectively), suggesting that BTM had a profound effect on the observation of osteoporosis therapy^14^. Some scholars believe that the combination of FRAX risk assessment tools and BTM will improve the prediction of OF risk^15^. Moreover, international guidelines have indicated that multi‐center data samples were necessary to expand BTM research‐related OF and to confirm their specific relationship^3^. It follows that BTM has great value in OF prediction and can be compared with BMD for prediction effect.

### *Analysis of the Significance of Bone Mineral Density, Aminoterminal Propeptide of Type I Procollagen, β‐Cross‐Linked C‐Telopeptide of Type I Collagen, and Molecular Fragment of Osteocalcin N Terminal in Older Women*

Our study investigated whether BMD and BTM could predict OF in a group of women with osteoporosis aged 60–90 years. There were 96 patients in the fracture group and 107 patients in the non‐fracture group. The results showed that the BMD in the fracture group was lower than in the non‐fracture group; the difference was statistically significant (*P* < 0.05), which indicated that the BMD in OF women was lower than in older women with osteoporosis alone. Therefore, bone mass loss in the OF women was greater; some published studies had similar findings^16^, ^17^.

PINP and β‐CTX were higher in the fracture group than in the non‐fracture group, and N‐MID in 70–90 year‐old patients was lower than for the non‐fracture group with statistically significant difference (*P* < 0.05); these results were similar to those of Tang (in Chinese)^18^. Because the bone metabolism of osteoporosis patients is in a high conversion state, all BMT are increasing; PINP and β‐CTX reflect biochemical markers of bone turnover. In female groups with OF, bone resorption is higher than bone formation, and N‐MID in fracture group may be lower than non‐fracture patients.

PINP is the decomposition of extended polypeptides in the metabolism of bone collagen cells, which reflects the synthesis rate of type I bone collagen and the state of new bone formation. PINP increases while bone conversion accelerates^19^. Shigdel *et al*. reported that postmenopausal women had higher PINP levels; their study indicated that the increase of PINP was correlated with the increase of cortical bone space, thickness reduction, and fracture probability of the hip (*P* < 0.05)^20^. A prospective cohort study by Yan *et al*. showed that PINP and CTX in a hip fracture group were significantly higher than those in a control group (*P* < 0.05) and were positively correlated with hip fracture (*OR* = 6.63 and 4.92 respectively)^21^. However, the age of this study was limited to 45–74‐year‐olds who were not all osteoporosis patients; their were results slightly different from our results. In our study, all the older women with osteoporosis had higher PINP and CTX in the fracture group (*P* < 0.05). The OF risk predicted by PINP was lower than for CTX, but the *OR* indicated by PINP was not statistically significant (*P* > 0.05). Another study showed that the vertebral fracture healing process could be judged through dynamic evaluation of PINP^22^.

N‐MID in the fracture group was lower than in the non‐fracture group (*P* < 0.05), which was not consistent with the theory. The BMD of the fracture group was lower and the bone conversion rate was higher than for the non‐fracture group. Obrant *et al*. reported that N‐MID and β‐CTX levels increased following fractures in women over 75 years old, but there was no statistical significance in comparing intra‐group N‐MID levels within various time endpoints of 2 years (*P* > 0.05), while β‐CTX levels were significantly increased (*P* < 0.05)^23^. After incurring OF, the bone formation process represented by N‐MID likely slows or even stops, and β‐CTX may be progressively increased, which indicates accelerated bone loss; therefore, the risk of OF recurrence increases in older women.

β‐CTX is a sensitive indicator of bone resorption that has made outstanding contributions to treatment monitoring and fracture prevention^5^. Our results suggested that β‐CTX in the fracture group was significantly higher than in the non‐fracture group (*P* < 0.05). Patients with high OF risk had higher bone conversion and stronger bone resorption ability; this result concurred with those of Lou *et al*.^24^. Another meta‐analysis showed a significant correlation between CTX and fracture risk without adjustment of BMD (*GR* = 1.18, 95% *CI* 1.05–1.34), and even higher risk of hip fracture (*GR* = 1.23, 95% *CI* 1.04–1.47)^25^. Furthermore, accelerating bone loss within 12 months after fragility fractures was associated with CTX, and early monitoring of β‐CTX was of great value for predicting the recurrence of fragility fractures^26^.

### *Analysis of the Levels of Alkaline Phosphatase, Calcium, and Phosphorus in Older Women*

There was no statistically significant difference in ALP, P, and Ca between the two groups (*P* > 0.05), which may be because levels of mineral salts such as P and Ca tend to be normal in patients with primary osteoporosis; this was consistent with findings by Balto *et al*.^27^. Serum ALP can reflect some bone ALP but cannot fully represent bone ALP indicators. However, a study by Mukaiyama *et al*. suggested that serum ALP in osteoporosis patients was higher and positively correlated with bone ALP (*P* < 0.05)^28^. The results of our study were negative, possibly due to the lack of a non‐osteoporosis group for statistical contrast. ALP is of great significance to the judgment regarding OF healing in older women when metabolic bone diseases caused by primary hyperparathyroidism are excluded^22^ for vertebral compression fractures, it is also strongly recommended that the efficacy of drug therapy be monitored^29^.

Bone mineral density (BMD) and bone turnover markers (BTM) in older women with osteoporosis, and to compare their predictive power for osteoporotic fractures (OF).

#### *Analysis of the Correlation of Osteoporotic Fractures with Bone Mineral Density and Bone Turnover Markers*

Correlation analysis showed that BMDL, BMDH, and β‐CTX were significantly correlated with the occurrence of OF (*OR* = −4.182, −6.929, and 7.572, respectively), and the differences were statistically significant (*P* < 0.05). The lower the bone density is, the higher the risk of fracture is. In addition, the higher the β‐CTX, the greater the risk of fracture. BMD is a protective factor for the occurrence of OF, but β‐CTX is a risk factor. BMD is the gold standard for the diagnosis of osteoporosis and has unique advantages in predicting the first fragility fracture for high‐risk individuals^30^. Unfortunately, it may miss out some people with clinical and epidemiological risk factors^31^; also, BMD is not specified in the project of the FRAX evaluation tool. β‐CTX may have serological fluctuations prior to changes in BMD, and high conversion rates mean increasing bone loss and fracture risk^32^. The present study results suggested that the absolute OR value of β‐CTX was larger, so β‐CTX was better at predicting OF risk. In fact, for older women with osteoporosis, regular monitoring of β‐CTX could be recommended.

PINP and N‐MID were positively correlated with the occurrence of OF (*OR* = 4.213 and 2.510, respectively), and the difference was not statistically significant (*P* > 0.05). The negative results may be caused by the focus on a single center and the low sample size in this study, and the serological indicators were unstable. The value of using PINP to predict OF has not been confirmed. Some scholars believe that PINP and OF are not correlated^33^, while others believe that they are positively correlated^34^. The report indicated that PINP may be more effective than CTX for predicting fractures^35^. Nguyen *et al*. reported that osteoporosis patients had higher levels of PINP and β‐CTX, but only β‐CTX was significantly correlated with BMD (*P* < 0.01)^36^. Eastell *et al*. believed that PINP could not be used to determine the amount of bone loss and predict fractures in individuals^37^. However, PINP and β‐CTX have an evident advantage when considering drug holidays for osteoporosis treatment^38^. Therefore, PINP and N‐MID currently lack specific clinical demonstrations to confirm their correlation with OF, which requires further research and in‐depth analysis.

### *Limitations*

This study is not without limitations. First, serological samples may be more statistically significant with more data. In addition, this was a single‐center clinical study, and partial bias existed in data measurement due to inappropriate operation, quality inspection, and other problems. BTM was also affected by dietary and circadian rhythms^39^. Therefore, we need a prospective, randomized controlled trial to further elucidate related issues.

### *Conclusion*

In conclusion, the prevalence of OF is increasing in older women, and many problems need to be solved urgently. BTM detection, including with PINP, β‐CTX, and N‐MID, has important clinical significance. Although a consensus has been reached regarding the advantages of using BMD to predict OF, there are still some deficiencies. The results of this study indicated that β‐CTX has more benefits over BMD in predicting OF. It recommends that BMD and β‐CTX should be measured in older women with osteoporosis, and appropriate management interventions should be adopted to avoid the occurrence of severe OF. Certainly, the use of PINP and N‐MID to predict OF occurrence remains to be discussed in future work, and a follow‐up multi‐center study will be conducted with an increased sample size.
